# Nerve Terminal Degeneration Is Independent of Muscle Fiber Genotype in SOD1^G93A^ Mice

**DOI:** 10.1371/journal.pone.0009802

**Published:** 2010-03-22

**Authors:** Dario I. Carrasco, Edyta K. Bichler, Kevin L. Seburn, Martin J. Pinter

**Affiliations:** 1 Department of Physiology, Emory University, Atlanta, Georgia, United States of America; 2 The Jackson Laboratory, Bar Harbor, Maine, United States of America; Brigham and Women's Hospital, Harvard Medical School, United States of America

## Abstract

**Background:**

Motor neuron degeneration in SOD1^G93A^ transgenic mice begins at the nerve terminal. Here we examine whether this degeneration depends on expression of mutant SOD1 in muscle fibers.

**Methodology/Principal Findings:**

Hindlimb muscles were transplanted between wild-type and SOD1^G93A^ transgenic mice and the innervation status of neuromuscular junctions (NMJs) was examined after 2 months. The results showed that muscles from SOD1^G93A^ mice did not induce motor terminal degeneration in wildtype mice and that muscles from wildtype mice did not prevent degeneration in SOD1^G93A^ transgenic mice. Control studies demonstrated that muscles transplanted from SOD1^G93A^ mice continued to express mutant SOD1 protein. Experiments on wildtype mice established that the host supplied terminal Schwann cells (TSCs) at the NMJs of transplanted muscles.

**Conclusions/Significance:**

These results indicate that expression of the mutant protein in muscle is not needed to cause motor terminal degeneration in SOD1^G93A^ transgenic mice and that a combination of motor terminals, motor axons and Schwann cells, all of which express mutant protein may be sufficient.

## Introduction

The SOD1^G93A^ transgenic mouse is a common model for studying motor neuron disease and ubiquitously expresses one (G93A) of many SOD1 protein mutations known to occur in about 20% of human cases of inherited motor neuron disease [1]. Previous studies have shown that expression of the mutated SOD1^G93A^ gene solely in neurons does not cause motor neuron disease [2]. This and other evidence suggest that toxic interactions between motor neurons and other cells all of which express the mutant SOD1 protein may be important for disease progression [3]–[5]. An important consideration is where to place emphasis in the search for such interactions. Recent evidence indicates that motor terminal degeneration in SOD1^G93A^ mice occurs long before motoneuron cell death in the spinal cord [6]–[8]. This evidence indicates that loss of motor unit function is the result of degenerative events in the periphery and not motor neuron cell death [9], [10]. Similar phenomena were reported earlier in a canine version of inherited motor neuron disease [11]–[13].

Motor neuron cellular partners that might contribute to motor terminal degeneration include myelinating Schwann cells and, at the neuromuscular junction (NMJ), muscle fibers and terminal Schwann cells (TSC). The results of an earlier study in which muscles were transplanted between SOD1^G93A^ and wild-type mice suggested that muscle may exert a toxic influence in SOD1^G93A^ mice [14]. Another study, however, reported that expression of mutant SOD1 protein in muscle does not contribute to the pathogenesis of SOD1^G93A^ mice [15]. The approach used in the latter study was to inhibit expression of mutated SOD1 protein in muscle but the results showed that the inhibition was not complete and leave open the possibility that expression in some or many muscle fibers may have been unaffected.

In order to clarify the role of muscle in determining motor terminal degeneration in SOD1^G93A^ mice, we transplanted whole muscles between SOD1^G93A^ and wild-type mice in the present study. The results showed that mutant SOD1-expressing muscles were not able to induce motor terminal degeneration in wildtype animals and that wildtype SOD1-expressing muscles were unable to prevent motor terminal degeneration in SOD1^G93A^ transgenic mice. The results thus demonstrate that the properties of the host animal and not the muscle transplant source determine whether degenerative changes at the NMJ are subsequently observed. Since the source of TSCs in transplanted adult muscles was not known with certainty, we examined this issue using transgenic mice with fluorescently-labeled TSCs [16]. Here we show that in the adult, all TSCs in regenerated muscle transplants are derived from the host. Our results indicate that pairings of SOD1-expressing Schwann cells, motor terminals and motor axons are sufficient to enable motor terminal degenerative changes in the SOD1^G93A^ mouse and that interactions with mutant SOD1 expressing muscle fibers do not play a role. Some of these results have been reported previously in abstract form [17].

## Results

### Wild-type motor neurons successfully innervate SOD1 MG transplants

**Figure 1:**
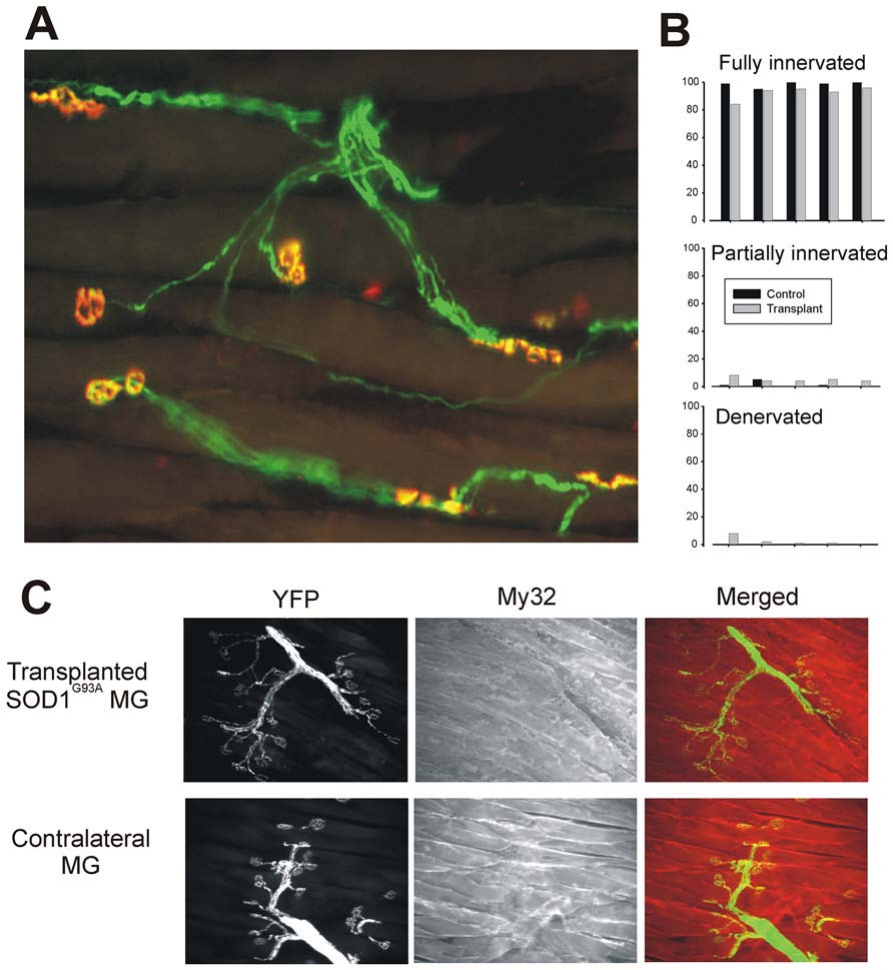
Wild-type motor neurons successfully reinnervate B6.SOD1 muscles. A. Low magnification view of an MG muscle from an B6.SOD1mouse transplanted into a wild-type animal 2 months earlier. The host B6.SOD1 animal expressed YFP in neuronal membranes (green) while ACHRs at endplates were labeled with α-bungarotoxin (α−Btx, red). The endplates in this view were all fully innervated by YFP-fluorescent motor terminals as indicated by yellow color. B. Bar charts show percentages of fully and partially innervated endplates and denervated endplates for transplanted and contralateral control MG muscles as determined by synaptophysin staining (not shown). Bar pairs correspond to the same animals. C. Low magnification views of YFP-labeled motor terminals and synapses (column 1), views of muscle fibers stained with an antibody against fast myosin (My32, column 2) and merged views (YFP, green) for sections taken from a transplanted MG muscle and a contralateral control MG from one animal. These panels demonstrate that fast muscle fibers regenerated in transplanted B6.SOD1 MG and were as completely innervated in transplanted B6.SOD1 MG muscle as in the contralateral MG muscle.

In this study, SOD1 animals on a B6 background (B6.SOD1) and wildtype B6 controls both possessed YFP-labeled axons to facilitate imaging [18]. MG muscles from B6.SOD1 donors were transplanted into wild-type hosts and recovered and analyzed for innervation status 2 months later. Sections taken from these muscles revealed the presence of many YFP-labeled axons that extended throughout an endplate band to form synaptic contacts on motor endplates labeled for acetylcholine (ACh) receptors with α−bungarotoxin (Figure 1A). Although not analyzed, the number of endplates in transplanted muscles appeared to be similar to contralateral, control muscles, and similar numbers of endplates were analyzed for innervation status (transplant 133±8, contralateral 144±4, N = 5 for both). The innervation status of muscle fibers in transplanted muscles was compared with the innervation status of the contralateral, control muscles by determining the extent to which endplates labeled for ACh receptors were occupied by synaptophysin-labeled nerve terminals. The results showed that transplanted B6.SOD1 muscles featured similar percentages of innervated and partially innervated muscle fibers relative to contralateral MG muscles (Figure 1B). The relatively small differences of innervation status were sufficient to produce a statistically significant association between innervation status and muscle source (transplant or control, p<0.01). The significance, however, depended upon comparisons within 2 animals in which the control muscles contained endplates that were 0% partially innervated and denervated and transplant muscles that had small but discrete percentages in these categories (5% on average or less). Factors other than genotype likely play a role in determining the small amounts of partial innervation and denervation in the transplanted muscles. Even in transplants of completely normal muscles, reinnervation is delayed by muscle fiber regeneration that follows after elimination of necrotic fibers [19], [20].

The results of several previous studies suggest that motor neurons which innervate fast type motor units are more susceptible to degeneration in SOD1^G93A^ mice [6], [21]. If such an effect is based on toxicity mediated by fast-type muscle fibers, then the overall successful innervation of B6.SOD1 transplanted muscle by wild-type motoneurons could be based on a failure of fast-type muscle fibers to regenerate in the transplanted muscles. However, sections of transplanted B6.SOD1 MG muscles showed that many innervated muscle fibers stained positively with an antibody for fast myosin and in this respect, these fibers were similar to those found in contralateral MG muscles (Figure 1C). In other sections from transplanted and contralateral control muscles, fewer muscle fibers were observed that did not stain positively for fast myosin (data not shown), consistent with the presence of a minority (ca. 6%) of slow muscle fibers in the mouse MG muscle [22]. These data demonstrate that B6.SOD1 MG muscles and motor endplates regenerate after being transplanted into wild-type animals and express different fiber types. Overall, wild-type motor neurons and motor terminals were able to innervate the transplanted B6.SOD1 MG muscles almost completely. Muscle fibers from B6.SOD1 mice are thus able to receive and maintain innervation from wild-type motor terminals.

**Figure 2:**
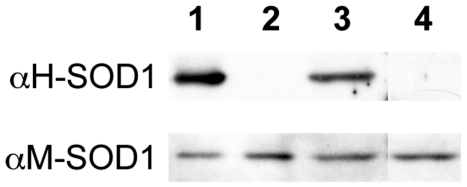
SOD1^G93A^ mutant protein is expressed in transplanted muscles. Western blots showing reactivity of antibodies against mouse SOD1 protein (αM-SOD1) and against human SOD1 protein (αH-SOD1) which is used to label the human mutant SOD1 protein. Lanes are as follows: 1. B6.SOD1 transgenic MG muscle; 2. Wild-type MG muscle; 3. MG muscle from B6.SOD1 transgenic transplanted 2 months earlier into a wild-type animal; 4. Control muscles were obtained from the ipsilateral limb (soleus).

Another explanation for the nearly complete innervation of B6.SOD1 MG transplants by wild-type motor neurons is that the regenerated fibers in transplants do not express the mutant SOD1 protein. Previous studies have shown, however, that adult muscles retain transgene expression following transplantation into immunocompatible hosts [23]. In order to confirm this in the present study, we performed Western analysis of SOD1 expression in MG muscle from an B6.SOD1 donor that had been transplanted into a wild-type host 2 months earlier. The results (Figure 2) confirm that expression of the mutated SOD1 protein is maintained in muscles transplanted from B6.SOD1 animals into wild-type animals. Moreover, these data demonstrate that the source of the muscle fibers in the transplant is the SOD1 donor animal

### Wild-type MG transplants do not prevent B6.SOD1 motor terminal degeneration

**Figure 3:**
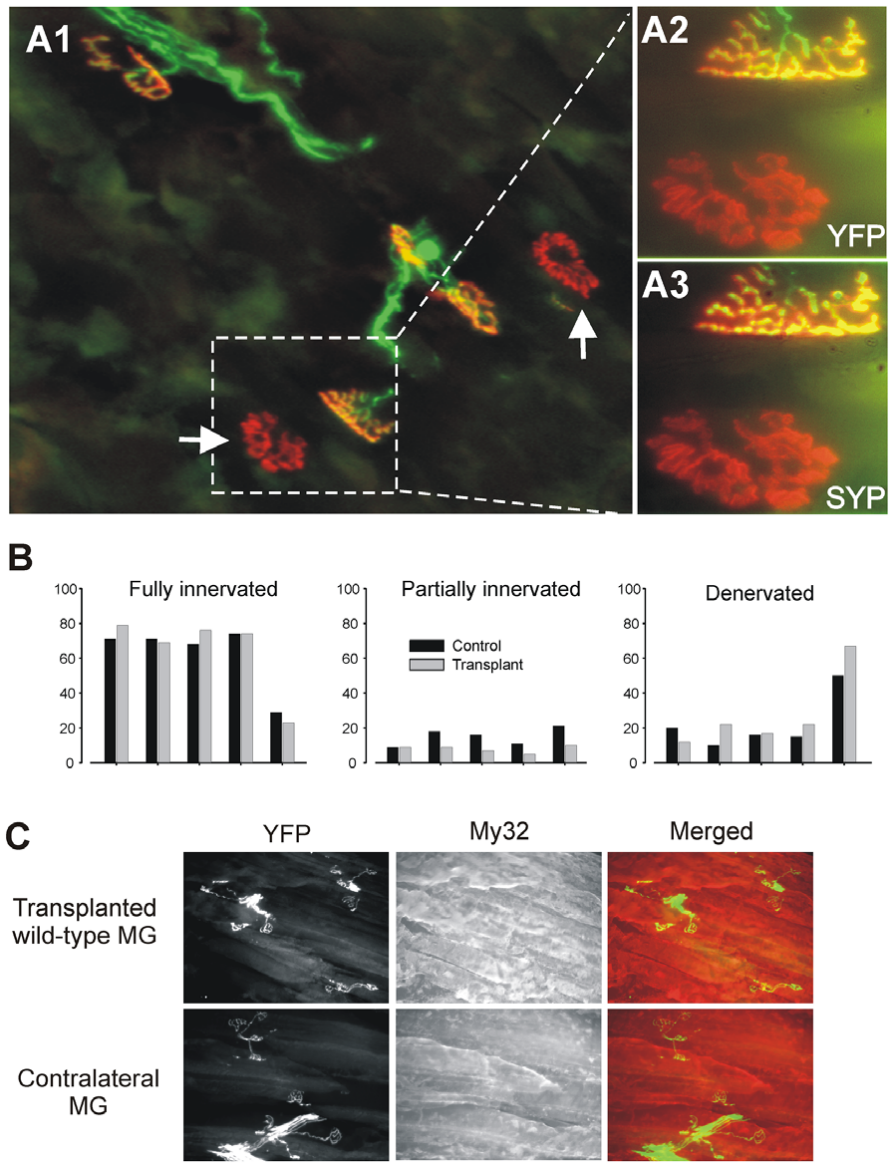
B6.SOD1 motor neurons fail to completely reinnervate wild-type muscles. A1. Low magnification view of wild-type muscle transplanted into an B6.SOD1 animal 2 months earlier. The host wildtype animal expressed YFP in neuronal membranes (green) while ACHRs are labeled with α−Btx (red). Denervated endplates are indicated by white arrows. A2–3. Higher magnification and rotated view of area indicated in A1 by broken lines. Panels illustrate complete occupation of upper endplate by nerve terminal (A2, YFP) and synaptophysin staining (A3, SYP) and complete denervation of lower endplate. B. Percentages of fully and partially innervated endplates and denervated endplates for transplanted and contralateral control MG muscles. Bar pairs correspond to the same animals. C. Low magnification views of YFP-labeled motor terminals and My32–stained muscle fibers show that fast muscle fibers regenerated in wild-type MG muscle transplanted into B6.SOD1 animals and showed similar levels of denervation.

In another series of experiments, wild-type MG muscles were transplanted into the legs of B6.SOD1 transgenic mice. In contrast with the transplantation of B6.SOD1 muscle into wild-type animals, an appreciable number of denervated and partially occupied endplates were encountered in the transplanted MG muscles (Figure 3A). The extent of NMJ abnormalities in the transplanted muscles, however, was similar to contralateral muscles. To quantify this observation, the innervations status of transplanted and contralateral control muscles was evaluated as described above. Two months after transplantation, we observed that the average percentages of innervated, partially innervated and denervated muscle fibers in the wild-type MG transplants did not differ significantly from percentages obtained from control, unoperated, MG muscles of B6.SOD1 mice (p>0.05, Figure 3B). As was the case in B6.SOD1 muscles transplanted into wild-type animals, the number of endplates in transplanted wildtype muscles did not appear to differ from contralateral, control muscles, and similar numbers were available for analysis (144±9 for transplants, 128±6 for control muscles, both N = 5). In addition, fiber type expression in wild-type MG muscles transplanted into B6.SOD1 animals was similar to contralateral MG muscles and was dominated by staining for fast myosin (Figure 3C).

The overall lack of difference of innervation status between transplanted and contralateral muscles could arise because some fibers in the transplanted wild-type muscles never became innervated. This possibility cannot be excluded since we did not use repeated *in vivo* imaging of endplates in transplanted muscles throughout the post-operative period. In one B6.SOD1 host animal, however, the MG muscle on the side contralateral to the transplant exhibited considerably less innervation than the remainder of the group, but this was matched by a similarly low percentage of fully innervated fibers on the transplanted side compared with muscles from other transgenic animals (Figure 3B). The overall side-to-side similarity of innervation status suggests that motor terminals in the transplanted wild-type muscle are subject to the same degeneration schedule as motor terminals on the contralateral side. Combined with the fact that most fibers (ca. 65%) in the transplants were found to be innervated in 4/5 experiments, these results suggest that the transplants were extensively innervated initially and that some fibers became denervated only later.

**Figure 4:**
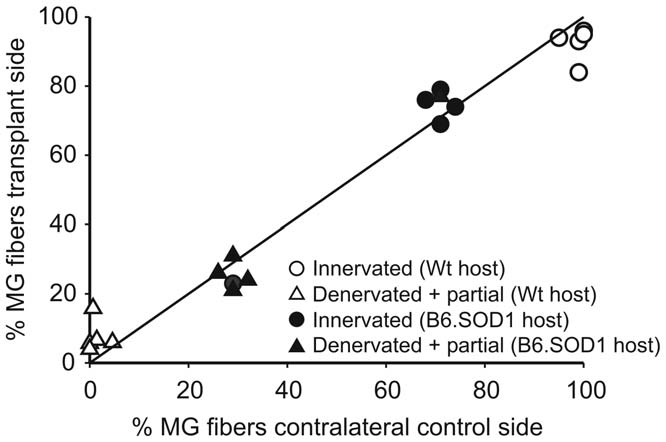
NMJ innervation status in muscle transplants is determined by the host. Panel shows a plot of percentage of innervated and of partially-innervated and denervated MG muscle fibers observed in MG muscle transplants versus the same percentages from contralateral control MG muscles. Symbols are defined by inset. Straight line in plot has a unitary slope. Data from all study animals are included in this plot. The association of innervation status between transplanted and control muscles suggests mutual dependence on the properties of the host.

Figure 4 summarizes innervation status and Table 1 provides group means for all transplant experiments. For both types of transplant experiment, there was an equivalence of innervation status between transplant and contralateral control muscle pairs. This observation indicates that innervation in transplanted and contralateral control muscles is affected by a common factor The most obvious common factor associated with transplanted and contralateral muscle pairs is the identity of the host and not the identity of the donor. This indicates that the properties of the transplant recipient determine innervation status in both transplanted and contralateral control muscles. By extension, this supports the conclusion that the properties of transplanted muscles have no effect on innervation status in either wild-type or B6.SOD1 transgenic mice.

**Table 1:** Group means of innervations status for contralateral control and transplanted muscles.

	Innervated		Partial		Dennervated	
Donor/host	Contralateral	Transplant	Contralateral	Transplant	Contralateral	Transplant
B6.SOD1/WT	99±1	93±2	1±1	5±1	0±0	2±1
WT/B6.SOD1	63±8	64±10	15±2	8±1	22±7	28±10

Values represents mean percentage ±1 SEM. N = 5 for all means.

### Terminal Schwann cells are derived from the transplant host

In addition to its relationship with the muscle fiber, the motor terminal is intimately covered by the terminal Schwann cell (TSC). Because of this close relationship, potentially toxic interactions with TSCs could be a factor in determining or influencing motor terminal degeneration in SOD1^G93A^ mice. The source of TSCs in transplanted muscles is not clear, however. Previously, it has been reported that only about half of TSCs are derived from the host when neonatal whole muscles are transplanted [24]. In order to determine the source of TSCs in the present experiments in adults, we performed MG muscle transplants between wild-type mice and transgenic mice that express EGFP under the direction of the human S100 calcium-binding protein promoter [16]. Because TSCs in these transgenic mice (B6.EGFP) are fluorescent, it is possible determine the source of TSCs after transplantation.

**Figure 5:**
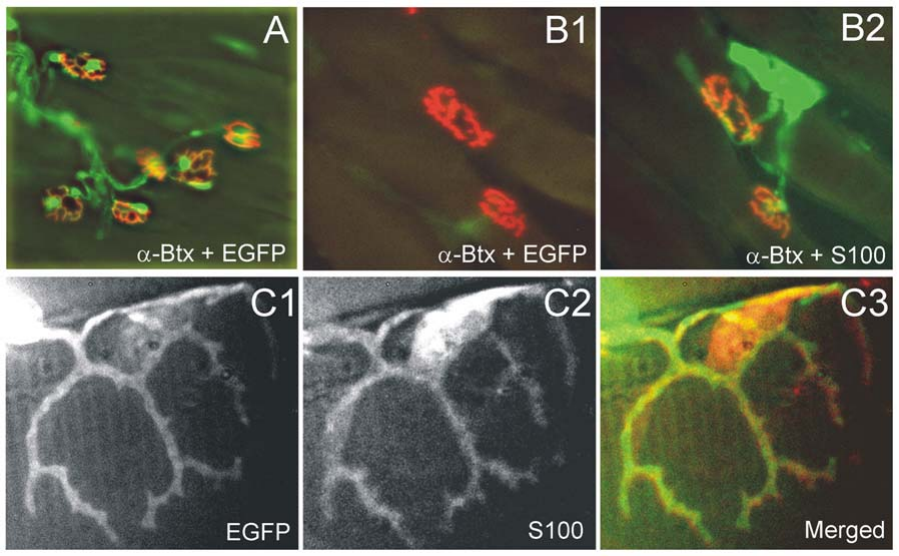
Terminal Schwann cells in transplanted adult muscle are derived from the host. A. Low magnification view of a wild-type MG muscle transplanted 2 months earlier into a transgenic mouse that expresses EGFP (green) under the direction of the S100 promoter. Endplate ACHRs are stained with α−Btx (red). This panel illustrates the general finding that all endplate staining colocalized with EGFP. B. EGFP fluorescence was absent at motor endplates in an MG muscle from an EGFP-expressing transgenic transplanted into a wild-type host (B1) but the same endplates stained positively for S100 (green, B2), demonstrating that terminal Schwann cells were derived from the host. C. To exclude the possible survival of wild-type TSCs after transplantation of wild-type muscle into the ECFP transgenic (C1), muscle sections were first labeled for S100 (C2) but all labeling was found to colocalize with EGFP fluorescence (C3) providing further evidence that wild-type TSCs did not survive transplantation.

Following a 2 month post-transplantation delay, we observed that all the MG muscles transplanted from wild-type mice into the B6.EGFP mice showed EGFP fluorescence at their NMJs (Figure 5A). In contrast, none of the NMJs in MG muscles from EGFP mice transplanted into wild-type mice exhibited EGFP fluorescence (Figure 5B). In order to determine whether TSCs were present but not EGFP fluorescent in the latter muscles, we labeled muscle sections with an antibody against the S100 protein and observed S100-positive staining at all nerve terminals (Figure 5B). This demonstrates that TSCs were present at these NMJs but did not contain the EGFP label. We also considered the possibility that some wild-type TSCs might survive the transplantation to B6.EGFP mice and express S100 but not EGFP. However, we observed that every instance of S100-positive staining co-localized with EGFP fluorescence (Figure 5C).

The co-localization of EGFP fluorescence and S100 staining at nerve terminals of MG muscles transplanted from wild-type mice into EGFP mice (Figure 5) indicates that all TSCs in the muscle transplants are provided by the host. Additional evidence indicates that donor TSCs do not survive the transplantation procedure. In a preliminary experiment, no evidence was detected of EGFP fluorescence or S100 antibody positive staining in an MG muscle transplanted 2 weeks previously from a wild-type mouse into an B6.EGFP mouse. However, both markers were present in the contralateral unoperated MG which was denervated at the time of the transplant surgery via ipsilateral sciatic nerve crush (data not shown).

## Discussion

One goal of the present study was to test the hypothesis that expression of the mutant SOD1^G93A^ protein in muscle is necessary for the appearance of motor terminal degeneration. To accomplish this, we used inbred mouse lines and transplanted muscles from B6.SOD1 transgenic mice into wild-type mice and from wild-type mice into B6.SOD1 transgenics. We found that NMJ innervation status was essentially equivalent between transplant and contralateral control muscle pairs in both types of transplant experiments (Figure 4). Since muscle pairs have the host in common, this association indicates that the properties of the host and not the donor determine NMJ innervation status. Thus, transplanted B6.SOD1 transgenic muscles successfully accept and retain innervation from wild-type motor neurons. Motor neurons in B6.SOD1 transgenic mice exhibit the same inability to either make or retain innervation on regenerated wild-type muscle fibers as do B6.SOD1 motor neurons innervating contralateral control muscles. These results indicate that mutant protein expression in muscle makes no contribution to denervation in SOD1 mice. Using behavioral tests, Miller et al [15] reached a similar conclusion after reducing expression of mutant protein in muscles of mice that ubiquitously express SOD1^G93A^. The reduction was incomplete, however, leaving open the possibility that reduced expression of mutant protein in muscle or a reduced number of expressing fibers is sufficient to cause the motor neuron disease phenotype. The observation that transplanted wild-type muscles do not inhibit the occurrence of muscle denervation in B6.SOD1 mice provides increased confidence that expression of the G93A SOD1 mutation in muscle is not necessary for the appearance of muscle denervation or the motor neuron disease phenotype.

An alternative explanation for the ability of wild-type motor neurons to innervate transplanted B6.SOD1 muscles is that the muscles did not express the mutated protein. This possibility was excluded by showing that B6.SOD1 muscle continues to express the mutated enzyme 2 months after transplantation into wild-type mice (Figure 2). This is consistent with the results of a previous study in which muscles continued expressing a transgene following transplantation into wild-type hosts [23]. The basis for these results likely resides in the mechanisms underlying recovery and regeneration following transplantation of whole muscles. Soon after transplantation, muscle fibers become ischemic and most appear to degenerate. Repopulation of muscle fibers is accomplished by activation and differentiation of satellite cells located within the original basal lamina [20]. Others have shown that host myoblasts do not invade transplanted muscles [24]. The source of regenerated muscle fibers in whole muscle transplants thus appears to be the transplanted muscle itself.

Another alternative idea is that MG motor terminal degeneration in the SOD1->Wt transplant group was absent because motor terminals were exposed for only 60 days (post-transplant interval) to SOD1 muscle. Although not identical, this time is comparable to the time MG motor terminals were exposed to SOD1 muscles in Wt->SOD1 transplant animals (80–85 days before receiving transplant of Wt muscle). However, only the latter group showed MG motor terminal degeneration while MG innervation in the former group was indistinguishable from normal. It is possible that the 2–3 week difference in exposure time is a factor in determining motor terminal degeneration. However, if the amount of exposure to potentially toxic muscle is a critical factor in determining degeneration, then one might reasonably expect to find that the unoperated sides of Wt->SOD1 transplant animals exhibit more extensive NMJ degeneration than the transplanted sides. In these cases, the unoperated sides were exposed to potentially toxic muscle for the entire lifetime of the animals (140–145 d) whereas the transplanted sides were exposed only for pre-transplant time intervals (80–85 d). Yet, as Figure 4 demonstrates, both sides showed essentially identical levels of denervation at 140–145 d. In the Wt->SOD1 group, we can not exclude that innervation of muscles that express mutant protein during earlier life intervals (0–80 d) sets in motion degenerative mechanisms that progress despite later innervation of wildtype muscle. However, our results do not provide clear support for the idea that the amount of exposure to mutant SOD1 muscle is critical for determining whether motor terminal degeneration occurs.

Selective regeneration of muscle fibers in transplanted muscles provides another possible explanation for the success of wild-type innervation of B6.SOD1 muscles. Although fiber number appears to recover in transplants of normal muscle [25], [26], we can not exclude that transgenic muscle fibers that may possess specific toxicity for motor terminals do not regenerate after transplantation. If such transgenic fibers exist, however, then the basis of the toxicity must involve features other than expression of the mutant B6.SOD1^G93A^ protein since fibers that did regenerate in transplants but did not cause motor terminal degeneration expressed the mutant protein. Other results suggest that B6.SOD1 motor neurons that innervate fast type motor units are more susceptible to degeneration in SOD1^G93A^ mice, an effect that could be based on toxicity of fast type muscle fibers [6], [10], [21]. Such type-specific selectivity is unlikely to be based upon intrinsic toxicity properties of fast muscle fibers since these muscle fibers in transplanted B6.SOD1 muscles were successfully re-innervated by wild-type motor neurons in a manner similar to contralateral, control wild type muscles.

The results also show that NMJ innervation status in wild-type muscles transplanted into B6.SOD1 mice is equivalent to the contralateral control side. This equivalence could arise because the transplants are initially completely reinnervated by B6.SOD1 motoneurons and subsequently become denervated, or because reinnervation is incomplete. It seems improbable that in every B6.SOD1 recipient, re-innervation failure in transplanted wild-type muscle would be nearly identical to active denervation on the control side. Since the majority of wild-type fibers were innervated on average 2 months after transplantation, it seems more likely that re-innervation of the wild-type transplant was fairly complete initially and that denervation occurred subsequently. If confirmed by sequential imaging, this would mean that B6.SOD1 motor neurons can support re-innervation of transplanted muscles initially but that degenerative changes eventually appear only after the synapse has matured and experiences increased activity.

### Terminal Schwann cells

Another issue addressed in the present study was the source of terminal Schwann cells (TSCs) in transplants, and the results show that in wild-type animals, the host provides TSCs located over motor terminals in muscles transplanted 2 months earlier. This indicates that adult TSCs, like muscle fibers, do not survive transplantation. The ability of TSCs to survive may differ in transplanted neonatal muscles where up to 50% of TSCs are reported to be derived from the host [24]. Re-populating TSCs are likely derived from host Schwann cells that invade the transplant muscle along with host nerve fibers in a manner similar to original innervation during embryonic development [27].

Since muscles transplanted from B6.SOD1 to wild-type animals all showed complete reinnervation and normal endplates, it seems reasonable to suppose that host-derived nerve terminal-TSC and Schwann cell-motor axon combinations were present. It also seems reasonable to infer that wild-type donor TSCs did not survive when wild-type muscles are transplanted into B6.SOD1 animals and are likely to be replaced by TSCs derived from the B6.SOD1 hosts. These ideas may have important implications for the conditions needed for either preventing or enabling motor terminal degeneration. Previously, Lino et al [2] showed that expression of the mutant SOD1^G93A^ protein in neurons alone (including motor neurons) was not sufficient to cause or greatly attenuate the motor neuron disease phenotype. The results of the present study show that expression of the mutant protein in muscle is similarly not sufficient to produce degeneration of motor terminals. However, when motor neuron-Schwann cell combinations are present in wild-type muscles transplanted into B6.SOD1 mice and both cell types likely express the SOD1^G93A^ mutation, nerve terminal degeneration proceeds in a manner indistinguishable from the contralateral, control side. Conversely, when the motor neuron-Schwann cell combination does not express the mutant SOD1^G93A^ protein, motor terminal degeneration does not proceed despite the presence of muscle that does express the mutant enzyme. Taken together, these results suggest that mutant protein expression by both motor neurons and Schwann cells is at least sufficient to enable motor terminal degeneration while expression in either motor neuron or muscle alone is not. It is possible that expression of mutant SOD1^G93A^ in TSCs and myelinating Schwann cells alone or in combination with motor neurons may be necessary for motor terminal degeneration.

Evidence has been reported previously that non-neuronal supporting cells (glial cells) play an important role in mediating the pathological changes observed the spinal cord of SOD1 mutant mouse [3]–[5], [10]. This evidence indicates that pathological changes in motor neuron cell bodies are not cell autonomous. The results of the present study extend these indications to include the motor axon, motor terminal and Schwann cells. This raises the interesting possibility that adverse relationships between glial cells and motor neurons in the spinal cord may be replicated in the periphery between motor terminals and TSCs. Since degeneration of motor terminals and peripheral motor axons precedes motor neuron cell death [6]–[8], adverse interactions between TSCs and motor terminals or between axons and myelinating cells could be of direct importance for determining loss of motor unit function in B6.SOD1 mice and conceivably trigger more widespread motor neuron degeneration. In normal animals, TSCs have important relationships with motor terminals that include the ability to induce sprouting [28] and mechanisms that influence synaptic release [29]. Evidence is also available that ablation of TSCs is associated with motor terminal degeneration following a delay [30]. These observations indicate the existence of crucial relationships between the TSC and the motor terminal that may turn out to be of importance for understanding how degenerative changes begin in the B6.SOD1 mouse and perhaps in human victims as well.

## Methods

### Animals

Strain background can have a powerful effect on phenotypic expression [31]. The background strain of mice used for many studies of the human SOD1 mutation is a B6SJL mixed hybrid strain [B6SJL-TgN(SOD1-G93A)1GUR] maintained by crossing transgene-positive male mice with B6SJL F1 hybrid females. This breeding strategy produces animals that are not genetically identical and thus random litter effects may significantly modify disease processes. To avoid this possible variation, we used an inbred congenic strain of mice B6.Cg-Tg(SOD1-G93A)1Gur/J carrying the G93A mutant form of the human SOD1 transgene [1]. In this paper, these mice arel referred to as B6.SOD1. These mice exhibit early changes in motor performance (ca. 50 days) but survive about 1 month longer than B6SJL SOD1 transgenic mice [32]. Congenic B6.SOD1G93A and C57BL/6J mice are mutually compatible donors because congenic mice have been backcrossed to C57BL/6J for 15 generations and are essentially genetically identical. Thus, immunosuppresive therapy was not performed in the present study.

In order to facilitate imaging, C57BL/6J (B6).Cg-Tg(Thy1-YFP)16Jrs/J mice (hereafter YFP16) [18] were crossed to B6.SOD1 mice to create double transgenics on the B6 background. These mice and YFP16 wildtypes were used for all transplant experiments involving the SOD1 mutation. For experiments involving terminal Schwann cell (TSC) fate after transplantation, B6.D2-Tg(S100B-EGFP)1Wjt/J mice were used (hereafter B6.EGFP). These mice express enhanced green fluorescent protein (EGFP) under the direction of the human S100 calcium-binding protein promoter [16] to produce fluorescent Schwann cells. All animals were acquired from The Jackson Laboratory (www.jax.org, Bar Harbor, ME).

### Transplant surgery

All transplants were performed under general anesthesia (ketamine 24 mg, xylazine 1.3 mg per ml)) For all transplant experiments, the right medial gastrocnemius (MG) muscle was carefully exposed and the tendons of origin and insertion and the MG nerve were severed. The MG muscle was then carefully separated from the lateral gastrocnemius muscle by cutting along the midline through the tendon shared by these two muscles. The excised MG was then placed into the bed created by the removal of MG muscle from the recipients. The tendons of origin and insertion of the transplanted MG were attached with a 10-0 suture to the remnants of tendons of the recipient MG muscle. The proximal stump of cut MG nerve of the recipient was placed in close proximity to the nerve stump of the MG transplant. The crural fasciae and skin incisions were closed using 6-0 monofilament suture. The animals were then returned to their cages for recovery and 2 full days of post-operative analgesics. Food and water were administered ad lib following recovery and animals were allowed to move freely in their cages.

Muscle transplantations were performed in B6.SOD1 animals aged 80–85 days. This age occurs after hindlimb gait disturbances can first be detected but well before readily apparent clinical symptoms appear [32]. Muscles were recovered from animals aged 140–145 days at which time symptoms begin to appear on average. All experiments were carried out in accordance with the Institutional Animal Care and Use Committee of Emory University.

### Western blotting

Muscles were homogenized on dry ice with a mortar and pestle and reconstituted at 10% (wt/vol) in RIPA buffer. Protein concentrations were determined by spectrophotometry via a BCA protein assay kit, and 20 ug samples of protein were fractionated by electrophoresis in 5% stacking and 15% resolving acrylamide gels and transferred to PVDF filters (Immobilon-P Transfer Membrane). Filters were then incubated with the affinity-purified antibody at 1∶500 dilution (Chemicon International #AB5482 Rabbit Anti-SOD1 mouse specific and #AB5480 Rabbit Anti-SOD1 human specific). Control and mutant extractions were loaded and run on the same gel.

### Immunolabeling

MG muscles were recovered from each limb and placed into 4% paraformaldehyde for 1 hr. Muscles were washed in a 0.1 M phosphate buffered solution (PBS) and incubated at 4°C overnight in PBS containing 20% sucrose for cryoprotection. Sections (50 µm thickness) were obtained using a Cyrostat (Leica). Motor endplate acetylcholine receptors (AChRs) were labeled with rhodamine conjugated α-bungarotoxin (Molecular Probes). Axons and motor nerve terminals were labeled with a mouse monoclonal antibody against the phosphorylated heavy fragment of neurofilament protein (SMI31, 1:400, Sternberger Monoclonal). Labeling was visualized using fluorescein-conjugated secondary antibody (1∶100, Jackson Immunoresearch Laboratories). Synaptic vesicles were labeled using a rabbit polyclonal antibody directed at synaptophysin (1∶100, Santa Cruz Biotechnology), and terminal Schwann cells (TSC) were labeled with a rabbit polyclonal antibody against S100 Ca^2+^-binding protein (Dako). Both synaptic vesicles and terminal Schwann cells were visualized using an AMCA-conjugated secondary antibody (1∶100, Jackson Immunoresearch Laboratories). Muscle fibers were labeled with a monoclonal antibody that recognizes the neonatal and all of the adult fast MyHC isoforms (MY-32, Sigma). Labeling was visualized with a rhodamine-conjugated secondary antibody (1∶100, Jackson Immunoresearch Laboratories).

### Imaging

Z-axis stacks of images of NMJs were obtained at sequential focal planes (0.5 µm separation) using an upright microscope equipped with a motorized stage (Leica). Stacks were deconvolved using a commercially available inverse filter algorithm (ImagePro). Illustrated images are flat plane projections obtained by summing deconvolved image stacks. Analysis consisted of evaluating NMJ innervation status for the extent to which presynaptic labeling overlaid postsynaptic labeling for AChRs in superimposed images. Three categories of innervation status were used as described previously [33]. Fibers were considered fully innervated if presynaptic labeling for synaptic vesicles completely covered the entire endplate area labeled for AChRs when images of vesicle and AChR labeling were superimposed. Fibers were considered partially innervated or denervated, if only parts or none, respectively, of the endplate labeled for ACh receptors area was occupied by the synaptic vesicles staining. For analysis, randomly selected fields of NMJs were first located at low magification. Then, all the NMJs in each field were categorized as described above.

### Statistics

Statistical comparisons of innervation status data were made between the transplanted (left) and the contralateral control (right) MG muscles. For analysis, two-way contingency tables (innervation status vs. muscle source (transplant or control) were used to determine whether statistically significant differences existed in the percentage of innervated and denervated endplates observed in the transplanted and control MG. To enable use of two-way tables, counts of partially innervated and denervated endplates were merged into one category characterized as not fully innervated. The Mantel-Haenszel test for 2 x 2 contingency tables to test for association between innervation and muscle source (side) in individual animals while controlling for possible effects of different animals. All analysis was performed with commercially available software (Systat Inc). Mean values are presented ±1 SEM.
